# Mental health literacy, self-regulatory fatigue, and attitudes toward aging among community-dwelling older adults with diabetes and their spouses: an actor–partner interdependence mediation model analysis

**DOI:** 10.3389/fpubh.2026.1943940

**Published:** 2026-10-20

**Authors:** Pingxia Yu, Shiwen Wu, Hua Zhou, Quan Wen

**Affiliations:** 1 School of Nursing, Chengdu Medical College, Chengdu, Sichuan, China; 2 Key Laboratory of Intelligent Medical Care and Elderly Health Management, Chengdu, Sichuan, China; 3 Department of Gastroenterology, Chengdu Eighth People’s Hospital, Chengdu, Sichuan, China; 4 Department of Traditional Chinese Medicine, Chengdu Eighth People's Hospital, Chengdu, Sichuan, China

**Keywords:** actor–partner interdependence mediation model, attitudes toward aging, diabetes mellitus, mental health literacy, older adults, self-regulatory fatigue, spouses

## Abstract

**Objective:**

This study aimed to examine the dyadic relationships among mental health literacy, ego depletion self-regulatory fatigue, and attitudes toward aging in community-dwelling older adults with diabetes and their spouses using an Actor–Partner Interdependence Mediation Model (APIMeM).

**Methods:**

A convenience sample of 221 dyads consisting of older adults with diabetes and their spouses was recruited from six communities in Chengdu, China, between October 2025 and April 2026. Data were collected using the General Information Questionnaire, the Chinese version of the Multicomponent Mental Health Literacy Measure (MMHL-CV), the Self-Regulatory Fatigue Scale (SRF-S), and the Attitudes to Aging Questionnaire (AAQ). An APIMeM was constructed using Mplus 8.3 to investigate actor and partner effects among mental health literacy, self-regulatory fatigue, and attitudes toward aging.

**Results:**

The mean mental health literacy scores of older adults with diabetes and their spouses were 12.12 ± 7.35 and 12.04 ± 7.23, respectively. The corresponding self-regulatory fatigue scores were 50.81 ± 15.72 and 49.42 ± 14.73, while the attitudes toward aging scores were 68.04 ± 21.32 and 68.48 ± 19.53, respectively. The Actor–Partner Interdependence Mediation Model (APIMeM) showed that mental health literacy was positively associated with both individuals’ own and their partners’ attitudes toward aging, and was negatively associated with both their own and their partners’ self-regulatory fatigue. In addition, self-regulatory fatigue was negatively associated with both individuals’ own and their partners’ attitudes toward aging (all *p* < 0.05).

**Conclusion:**

Mental health literacy, self-regulatory fatigue, and attitudes toward aging were significantly associated within community-dwelling older adults with diabetes and their spouses. Healthcare professionals should consider patients and their spouses as a dyadic health unit when designing comprehensive psychological support programs. Family-centered interventions may be associated with better psychological well-being and favorable healthy and positive aging.

## 1 Introduction

Type 2 diabetes mellitus is one of the most prevalent chronic metabolic diseases worldwide and represents a major public health challenge among older adults (1). The prevalence of diabetes increases substantially with age; epidemiological evidence indicates that approximately one-third of older adults in China are affected by diabetes (2). Due to the chronic and progressive nature of the condition, older adult patients often experience negative emotions, which reduce treatment compliance and self-management capabilities, thereby negatively influencing their attitudes toward aging (3). Attitudes toward aging—defined as older adults’ subjective experience of their own aging process—comprise both positive and negative components (4) and serve as a malleable factor significantly influencing health-promoting behaviors (5).

In community settings, diabetes management is rarely an individual endeavor; it is deeply embedded in the spousal relationship. Spouses often serve as primary caregivers and are themselves at a life stage susceptible to chronic diseases. The prolonged stress of caregiving and the shared lifestyle changes required for diabetes management can adversely affect the spouse’s physical and mental health, as well as their own attitudes toward aging. Consequently, patients and their spouses exhibit marked interdependence in illness management, suggesting that one partner’s psychological attributes may influence the other’s outcomes.

Mental health literacy, defined as beliefs pertaining to the identification, management, and prevention of mental health problems (6), plays a pivotal role in this dyadic context. Prior research indicates that higher mental health literacy facilitates shared positive illness perceptions (7). According to the dyadic coping framework, a spouse with high mental health literacy is better equipped to provide effective emotional support and engage in positive communication. This supportive behavior not only improves their own psychological state (actor effect) but also alleviates the patient’s psychological distress, potentially fostering more positive attitudes toward aging in the partner (partner effect).

However, the prolonged demands of chronic disease management may gradually deplete psychological resources, leading to self-regulatory fatigue. Self-regulatory fatigue is a sustained state characterized by reduced self-regulatory capacity and increased subjective fatigue (8, 9). Based on the Strength Model of Self-Control, individuals with low mental health literacy may lack the cognitive strategies to effectively manage disease-related stress, leading to a faster depletion of self-regulatory resources (i.e., higher self-regulatory fatigue). Conversely, high self-regulatory fatigue can impair an individual’s ability to maintain optimism, resulting in more negative attitudes toward aging. Therefore, self-regulatory fatigue may serve as a critical mechanism linking mental health literacy to attitudes toward aging.

Despite these theoretical links, the majority of existing research has concentrated on individual-level unidirectional effects, with limited attention to the patient–spouse dyad. To address this gap, the present study examined community-dwelling older adult patients with diabetes and their spouses. Unlike previous studies that merely establish an Actor–Partner Interdependence Mediation Model (APIMeM) (10) without specific predictions, this study proposes the following hypotheses: First, higher mental health literacy will be positively associated with positive attitudes toward aging for both patients and spouses (H1: Actor Effects). Second, higher mental health literacy in one partner will be positively associated with positive attitudes toward aging in the other partner (H2: Partner Effects). Third, self-regulatory fatigue will mediate the relationship between mental health literacy and attitudes toward aging (H3: Mediation). Finally, this mediating effect will manifest through both actor pathways and partner pathways (H4: Dyadic Mediation).

## 2 Subjects and methods

### 2.1 Subjects

Using convenience sampling, 221 couples consisting of older adult patients with diabetes and their spouses from six communities in Chengdu, China, were selected as study participants between October 2025 and April 2026.

Patient inclusion criteria: (1) Meet the diagnostic criteria outlined in the 2020 Chinese Guidelines for the Prevention and Treatment of Type 2 Diabetes (11); (2) Age ≥60 years; (3) Married with a living spouse; (4) Permanent residents of the community who have lived in their current residence for ≥6 months; (5) Normal cognitive function, able to understand and follow instructions; (6) Informed consent and voluntary participation in this study.

Patient exclusion criteria: (1) Patients with cognitive impairment, psychological disorders, or psychiatric disorders; (2) Patients with severe complications or life-threatening conditions, defined as unstable cardiovascular or cerebrovascular events (e.g., acute myocardial infarction, stroke within the past 6 months), end-stage renal disease requiring dialysis, or advanced malignancies.

Criteria for inclusion of spouses: (1) The spouse is the patient’s primary caregiver and has lived with the patient for more than 6 months; (2) The spouse has given informed consent and voluntarily agreed to participate in this study; (3) The spouse has no hearing or vision impairments and possesses good comprehension and communication skills.

Criteria for Excluding Spouses: (1) Those with cognitive impairments, psychological disorders, or mental illnesses; (2) Those with severe complications or suffering from serious, life-threatening illnesses, defined as unstable cardiovascular or cerebrovascular events (e.g., acute myocardial infarction, stroke within the past 6 months), end-stage renal disease requiring dialysis, or advanced malignancies.

According to the statistical requirements for multivariate analysis, the sample size should be 5 to 10 times the number of independent variables. This study included 27 independent variables; taking into account a 20% non-response rate, the study required a sample size of 169–338 pairs. The final analytical sample consisted of 221 dyads.

This study has been approved by the Ethics Committee of Chengdu Eighth People’s Hospital (Approval No.: 2025-CBYEC-001). All participants have provided informed consent and voluntarily participated in this study.

### 2.2 Survey tools

#### 2.2.1 General information questionnaire

The scale used in this study was self-developed by the researchers based on a literature review. The patient section comprised demographic and clinical variables, including gender, age, education level, average monthly household income, monthly treatment costs, health insurance type, diabetes duration, complications, glycemic control over the past 6 months, daily self-monitoring frequency, family history, and medication regimen. The spouse section comprised gender, age, education level, current comorbidities, daily caregiving hours, and total caregiving duration.

#### 2.2.2 Multicomponent mental health literacy measure-Chinese version (MMHL-CV)

The scale was originally developed by Jung et al. (12). For this study, we used the Chinese version, which was translated, culturally adapted, and validated by Ming et al. (13). The scale comprises three dimensions (knowledge, beliefs, and resources) with 22 items rated on a 5-point Likert scale. In the knowledge dimension, which consists of positively worded items, responses of “Agree” or “Strongly Agree” are scored 1, and all other responses 0. The Belief subscale consists of reverse-scored items, with “Disagree” and “Strongly Disagree” coded as 1 and all other responses as 0. The Resources subscale comprises true/false items, where a “Yes” response is scored 1 and “No” 0. Total scores range from 0 to 22, with higher scores indicating greater mental health literacy. The Cronbach’s α coefficient was reported as 0.80 in the original validation study. In this study, the coefficients were 0.945 and 0.942 for the diabetic patient and spouse subsamples, respectively. Confirmatory factor analyses for both the patient and spouse groups indicated a good fit of the three-factor model, with all indices meeting the recommended criteria (patients: *χ*^2^/df = 1.591, CFI = 0.947, TLI = 0.941, RMSEA = 0.052, SRMR = 0.042; spouses: *χ*^2^/df = 1.287, CFI = 0.972, TLI = 0.969, RMSEA = 0.036, SRMR = 0.055), demonstrating that the three-factor structure of the MMHL-CV is highly applicable to both groups.

#### 2.2.3 Self-regulatory fatigue scale (SRF-S)

The scale, initially developed by Nes et al. (14) and cross-culturally adapted for Chinese use by Wang et al. (15), was administered to evaluate self-regulatory fatigue among patients and their spouses. The scale comprises 16 items distributed across three dimensions: cognitive control (items 1–3, 5, 11, and 13), emotional control (items 7, 9, 12, 14, and 15), and behavioral control (items 4, 6, 8, 10, and 16). The scale employs a 5-point Likert response format (1 = “Strongly Disagree” to 5 = “Strongly Agree”). Five items (1, 2, 5, 9, and 14) are reverse-scored. Summing all items yields a total score between 16 and 80, with higher scores indicating more severe self-regulatory fatigue. The Cronbach’s α coefficient was reported as 0.84 in the original validation study. In this study, the coefficients were 0.942 and 0.934 for the diabetic patient and spouse subsamples, respectively.

#### 2.2.4 Attitudes to aging questionnaire (AAQ)

Originally developed by Laidlaw et al. (4) and subsequently adapted for Chinese populations by Huang et al. (16). The scale was used to assess patients’ and their spouses’ attitudes toward aging. The scale consists of 24 items distributed equally across three dimensions: psychosocial loss, physical changes, and psychological gains (8 items per dimension). The scale employs a 5-point Likert response format (1 = “completely incorrect” to 5 = “completely correct”), and items in the psychosocial loss dimension are reverse-coded. Summing all items yields a total score between 24 and 120, with higher scores reflecting more favorable aging attitudes. The Cronbach’s α coefficient was reported as 0.827 in the original validation study. In the present sample, the Cronbach’s α coefficients were 0.943 for older adult patients with diabetes and 0.928 for their spouses.

### 2.3 Data collection methods

All investigators received standardized training prior to the survey. They explained the study purpose and content to both patients and their spouses and obtained written informed consent. Questionnaires were then distributed, with each participant assigned a unique identification number to ensure accurate data matching within dyads. Upon completion, the questionnaires were collected on-site and reviewed item by item for missing responses; any omissions or inconsistencies were promptly verified with respondents and corrected. A total of 234 questionnaires were distributed to patients and 234 to their spouses, with 221 valid responses returned from each group, yielding a valid response rate of 94.4%.

### 2.4 Statistical analysis

Statistical analyses were conducted using SPSS 26.0 and Mplus 8.3. Categorical variables were presented as frequencies (n) and percentages (%). Normally distributed continuous variables were expressed as mean ± standard deviation (SD). Between-group comparisons were performed using paired t-tests or Wilcoxon signed-rank tests, as appropriate. Pearson correlation analysis was performed to examine bivariate associations among the study variables. Using Mplus 8.3, an actor–partner interdependence model (APIM) was constructed based on dyadic data to explore the relationships among mental health literacy, self-regulatory fatigue, and attitudes toward aging in older adult patients with diabetes and their spouses. The Bootstrap method with 5,000 resamples was applied to test mediating effects, with statistical significance set at *α* = 0.05.

## 3 Results

### 3.1 Participant characteristics

A total of 221 dyads of older adults with diabetes and their spouses were included in this study. The patients with diabetes were aged 60–83 years (mean age: 69.87 ± 5.24 years), while their spouses were aged 55–87 years (mean age: 69.88 ± 6.74 years). The remaining demographic and clinical characteristics are presented in Tables 1, 2.

**Table 1:** Baseline characteristics of older adults with diabetes (*n* = 221).

Variable	Classification	*n*	Percentage (%)
Gender	Male	123	55.7
	Female	98	44.3
Patients’ educational attainment	Illiterate	30	13.6
	Elementary School	57	25.8
	Middle School	59	26.7
	High School	43	19.5
	College and Above	32	14.5
Monthly per capita household income	<1,000 yuan	40	18.1
	1,000–3,000 yuan	47	21.3
	3,001–5,000 yuan	57	25.8
	>5,000 yuan	77	34.8
Health insurance	Yes	187	84.6
	No	34	15.4
Duration of diabetes	<5 years	43	19.5
	5–10 years	101	45.7
	>10 years	77	34.8
Diabetes complications	Yes	122	55.2
	No	99	44.8
Blood sugar control over the past 6 months	Well-controlled	9	4.1
	Moderately controlled	67	30.3
	Poorly controlled	73	33.0
	Unclear	72	32.6
Frequency of blood glucose monitoring	Daily	55	24.9
	Weekly	51	23.1
	Once a month or less	54	24.4
	Never	61	27.6
Family history of diabetes	Yes	85	38.5
	No	136	61.5
Administration	Insulin Therapy	68	30.8
	Oral Medication Therapy	34	15.4
	Combination Therapy with Insulin and Oral Medications	64	29.0
	Other	55	24.9
Monthly treatment costs	<500 yuan	36	16.3
	500–1,000 yuan	99	44.8
	>1,000 yuan	86	38.9

**Table 2:** Baseline characteristics of spouses of older adults with diabetes (*n* = 221).

Variable	Classification	*n*	Percentage(%)
Gender	Male	98	44.3
	Female	123	55.7
Age	<60 years	13	5.9
	60–70 years	101	45.7
	71–80 years	95	43.0
	>80 years	12	5.4
Educational background	Illiterate	48	21.7
	Elementary School	47	21.3
	Middle School	47	21.3
Current medical conditions	High School	32	14.5
	College and Above	47	21.3
	No chronic diseases	47	21.3
	High blood pressure	65	29.4
	Diabetes	34	15.4
	Cardiovascular and cerebrovascular diseases	27	12.2
	Other	48	21.7
Daily patient care time	<8 h	48	21.7
	8–16 h	63	28.5
	>16 h	110	49.8
Total caregiving duration	<50 h	58	26.2
	50–100 h	100	45.3
	101–150 h	44	19.9
	>150 h	19	8.6

### 3.2 Comparison of scores between patients and spouses

Table 3 presents the scores.

**Table 3:** Comparison of mental health literacy, self-regulatory fatigue, and attitudes toward aging between patients and spouses (mean ± SD).

Variable	*n*	Mental health literacy	Self-regulatory fatigue	Attitudes toward aging
Patients	221	12.12 ± 7.35	50.81 ± 15.72	68.04 ± 21.32
Spouses	221	12.04 ± 7.23	49.4 ± 14.73	68.48 ± 19.53
*t*		0.111	0.965	−0.226
*p*		0.912	0.335	0.822

### 3.3 Correlations among mental health literacy, self-regulatory fatigue, and attitudes toward aging

Pearson correlation results are shown in Table 4.

**Table 4:** Correlation matrix among study variables (*r*).

Variable	①	②	③	④	⑤	⑥
① Patient mental health literacy	1					
② Patient self-regulatory fatigue	−0.649**	1				
③ Patient attitudes toward aging	0.630**	−0.701**	1			
④ Spouse mental health literacy	0.527**	−0.521**	0.580**	1		
⑤ Spouse self-regulatory fatigue	−0.516**	0.575**	−0.589**	−0.588**	1	
⑥ Spouse attitudes toward aging	0.628**	−0.586**	0.662**	0.595**	−0.580**	1

**p < 0.01.

### 3.4 Common method bias test

Common method bias was assessed using Harman’s single-factor test. The first unrotated factor accounted for 27.187% of the total variance, which was below the recommended threshold of 40%, indicating that common method bias was not a serious concern in the present study and was unlikely to substantially influence the study findings.

### 3.5 Actor-partner interdependence mediation model

Using patients’ and spouses’ mental health literacy as predictors, self-regulatory fatigue as mediator, and attitudes toward aging as outcomes, an actor–partner interdependence mediation model was estimated with maximum likelihood. Because the model was saturated (df = 0), fit indices were *χ*^2^ = 0.000, RMSEA = 0.000, CFI = 1.000, TLI = 1.000, SRMR = 0.000. As expected for a saturated model, these values are mathematically determined and do not provide evaluative information regarding model fit. Consequently, the results were interpreted based primarily on the significance of the path coefficients.

#### 3.5.1 Actor effects

Mental health literacy of both patients and spouses was positively related to their own attitudes toward aging (*β*patient = 0.206, *p* < 0.003; *β*spouse = 0.242, *p* < 0.001). Mental health literacy was negatively related to own self-regulatory fatigue (*β*patient = −0.519, *p* < 0.001; *β*spouse = −0.437, *p* < 0.001), and self-regulatory fatigue was negatively related to own attitudes toward aging (*β*patient > = −0.384, *p* < 0.001; *β*spouse = −0.195, *p* = 0.002).

#### 3.5.2 Partner effects

Mental health literacy of patients and spouses was positively related to each other’s attitudes toward aging (*β*patient → spouse = 0.300, *p* < 0.001; *β*spouse → patient = 0.180, *p* = 0.003) and was negatively related to each other’s self-regulatory fatigue (*β*patient → spouse = −0.285, *p* < 0.001; *β*spouse → patient = −0.247, *p* < 0.001). Self-regulatory fatigue of both was negatively related to each other’s attitudes toward aging (*β*patient → spouse = −0.153, *p* = 0.032; *β*spouse → patient = −0.156, *p* = 0.011). Consistent with the APIM framework for distinguishable dyads, the model allowed for correlated residuals. Specifically, significant correlations were observed between patients’ and spouses’ self-regulatory fatigue (*r* = 0.288, *p* < 0.001) and attitudes toward aging (*r* = 0.255, *p* < 0.001) (see Figure 1).

**Figure 1:**
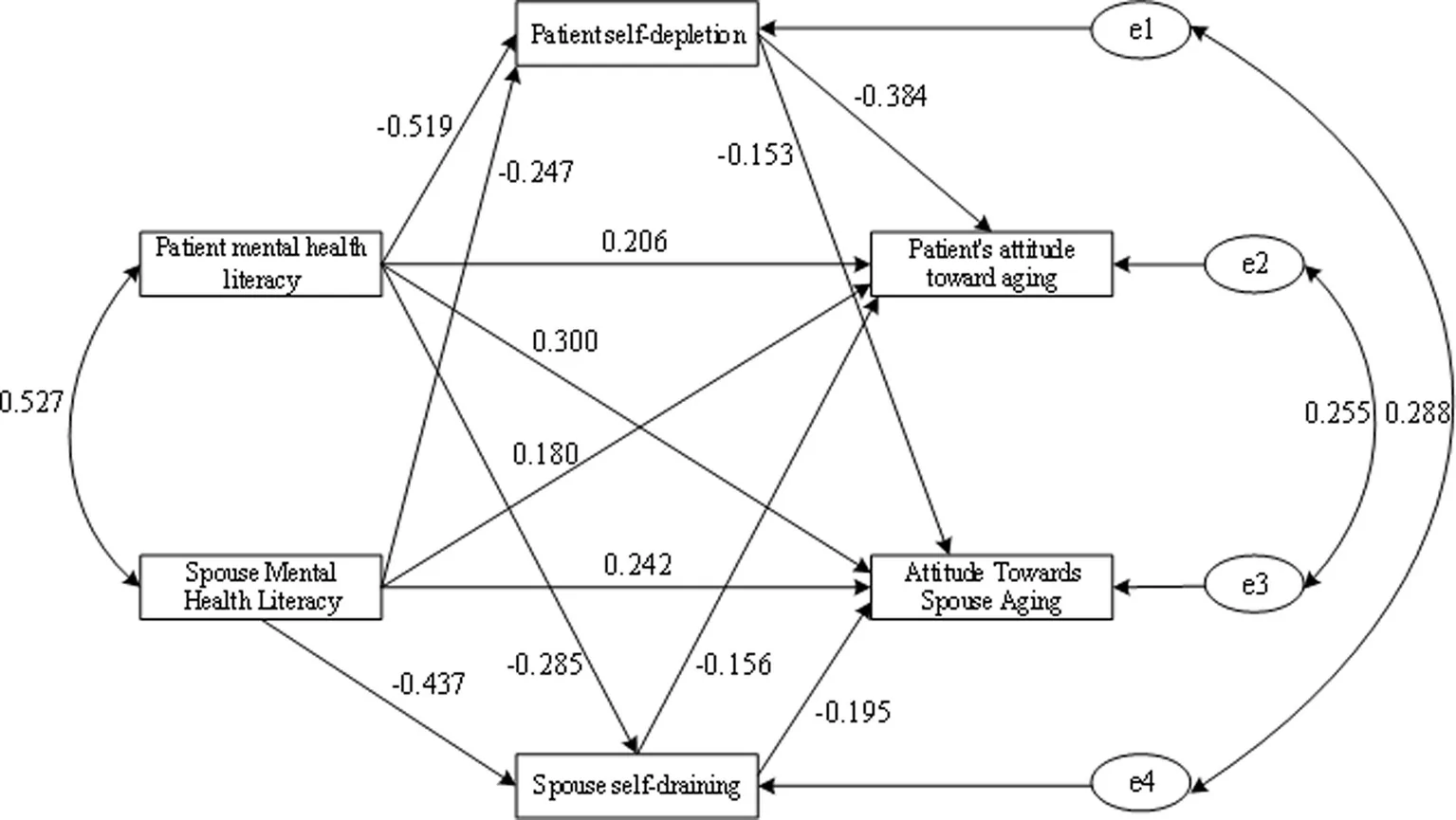
Standardized actor–partner interdependence mediation model of mental health literacy, self-regulatory fatigue, and attitudes toward aging in older adult patients with diabetes and their spouses.

Bootstrap confidence intervals (95% CI) for eight specific indirect paths did not contain zero, indicating all mediation effects were statistically significant (Table 5). Thus, self-regulatory fatigue mediated the actor–partner effects between mental health literacy and attitudes toward aging across both partners.

**Table 5:** Standardized actor–partner mediation effects of mental health literacy and self-regulatory fatigue on attitudes toward aging.

Mediation path	*β*	SE	*z*	95% CI	Conclusion
Patient MHL → Patient SRF → Patient AA	0.199	0.042	4.757	0.132 ~ 0.286	Significant
Spouse MHL → Spouse SRF → Spouse AA	0.085	0.031	2.783	0.033 ~ 0.153	Significant
Patient MHL → Spouse SRF → Spouse AA	0.056	0.022	2.566	0.021 ~ 0.109	Significant
Spouse MHL → Patient SRF → Patient AA	0.095	0.029	3.254	0.046 ~ 0.162	Significant
Patient MHL → Spouse SRF → Patient AA	0.045	0.022	2.005	0.011 ~ 0.101	Significant
Spouse MHL → Patient SRF → Spouse AA	0.038	0.019	1.939	0.006 ~ 0.083	Significant
Patient MHL → Patient SRF → Spouse AA	0.079	0.04	1.991	0.007 ~ 0.164	Significant
Spouse MHL → Spouse SRF → Patient AA	0.068	0.028	2.461	0.091 ~ 0.129	Significant

β = indirect effect; 95% CI excluding 0 indicates significance. MHL = mental health literacy; SRF = self-regulatory fatigue; AA = attitudes toward aging.

## 4 Discussion

### 4.1 Levels of mental health literacy, self-regulatory fatigue, and attitudes toward aging

In this sample, the mean scores for mental health literacy were 12.12 ± 7.35 for patients and 12.04 ± 7.23 for spouses. Jiang et al. (17) reported that access to mental health knowledge and services in China is insufficient, partly due to stigma associated with psychological problems in traditional culture. Consequently, older adult diabetic patients tend to avoid psychological distress and focus on physical symptoms, neglecting mental health. Moreover, limited channels for acquiring mental health knowledge further constrain their ability to recognize and manage psychological issues (18). Ren et al. (19) confirmed that mental health literacy interventions were linked to improved knowledge. Self-regulatory fatigue scores were 50.81 ± 15.72 for patients and 49.42 ± 14.73 for spouses, which are comparable to the findings of Meng and Lang (20). This may be related to the challenges associated with diabetes, such as a prolonged disease course and multiple complications. These factors necessitate sustained self-control, which may deplete self-regulatory resources and potentially contribute to anxiety or depressive symptoms. The scores for attitudes toward aging were 68.04 ± 21.32 for patients and 68.48 ± 19.53 for spouses, which are lower than those reported by Pan et al. (21) among patients undergoing maintenance hemodialysis. This discrepancy may be attributed to the more predictable treatment routines and better psychological adaptation observed in hemodialysis patients. In contrast, patients with diabetes face disease uncertainty and glycemic variability, which may, to some extent, exacerbate negative perceptions of aging.

### 4.2 Actor and partner effects

#### 4.2.1 Mental health literacy, self-regulatory fatigue, and aging attitudes exhibited significant actor effects in older adults with diabetes and their spouses

Mental health literacy was positively associated with one’s own attitudes toward aging. This suggests that individuals with lower mental health literacy experience higher self-regulatory fatigue, leading to helplessness and negative aging evaluations. Mental health literacy was negatively associated with own self-regulatory fatigue: those with lower literacy, under long-term self-management and caregiving pressure, have persistent resource depletion, leading to avoidance behaviors and increased fatigue. Therefore, community healthcare providers should enhance attention to both partners’ mental health literacy and conduct psychoeducational programs. Self-regulatory fatigue was negatively associated with own attitudes toward aging, resembling Segel-Karpas et al. (22) higher depletion correlates with more negative aging attitudes. During treatment, patients remain in a sustained self-control state, resulting in anxiety/depression and negative attitudes (23). Spouses, bearing caregiving responsibilities, must suppress negative emotions while facing their own chronic disease risks, exacerbating psychological resource exhaustion and negative aging attitudes. Studies on positive psychology suggest that positive psychological resources buffer self-control depletion and promote positive aging attitudes (24). Thus, community providers should design multilevel (individual, couple, community) ego-depletion management programs to reduce psychological burden and foster positive attitudes.

Both older patients with diabetes and their spouses showed that their mental health literacy was negatively associated with the other’s self-regulatory fatigue and was positively associated with the other’s aging attitudes.

Mental health literacy of each partner was negatively associated with the other’s self-regulatory fatigue and was positively associated with the other’s attitudes toward aging. In older adult diabetic families, mental health literacy can cross individual boundaries to influence the partner’s depletion and aging attitudes. In the community, patients and spouses are each other’s primary support. Patients with higher literacy are better at disease management and health-related knowledge, which alleviates caregiving burden and reduces passive psychological resource consumption in spouses, thus lowering self-regulatory fatigue. Conversely, spouses with low literacy may lack ability to recognize the patient’s psychological issues and fail to provide emotional support, allowing negative emotions to accumulate and worsen the patient’s depletion (25). Meanwhile, when supportive spouses themselves experience depletion, their negative emotions lack outlets and may transform into negative aging attitudes (26). Negative attitudes are not isolated; they can be transmitted through daily interactions and emotional contagion, mutually reinforcing negative perceptions and trapping both in a vicious cycle (27). Therefore, healthcare providers should treat the dyad as a health community, guide them to recognize each other’s emotional signals, and promote effective communication to achieve positive aging attitudes in disease management.

#### 4.2.2 Self-regulatory fatigue mediated the relationship between mental health literacy and aging attitudes in older patients with diabetes and their spouses

Self-regulatory fatigue significantly mediated the relationship between mental health literacy and attitudes toward aging in both actor and partner pathways. This suggests that mental health literacy, as a positive psychological resource, is not only directly associated with more favorable attitudes but also indirectly related to them through its association with lower self-regulatory fatigue. This aligns with the strength model of self-control: self-control consumes limited psychological resources; below a critical threshold, subsequent emotional and behavioral regulation declines (28). Mental health literacy, as cognitive resource, helps individuals recognize and cope with distress, reducing depletion for both, thereby improving aging attitudes. Thus, community providers should strengthen interventions targeting self-regulatory fatigue – e.g., simplifying disease management protocols and providing individualized guidance – to reduce self-control load and enhance positive aging attitudes for both partners.

### 4.3 Implications and limitations

This study examined the interrelationships among mental health literacy, self-regulatory fatigue, and attitudes toward aging in diabetic patients and spouses, confirming that self-regulatory fatigue mediates both actor and partner effects. The findings suggest that healthcare providers should view the dyad as a “health community” and develop integrated psychosocial support programs to reduce depletion and promote positive aging attitudes. However, several limitations should be noted. First, the cross-sectional design precludes causal inference and does not allow for the control of potential confounding factors. Second, strict exclusion criteria regarding mental health status and sensory impairments may have introduced selection bias, resulting in a sample with fewer psychological issues and less physical frailty than the general community. Consequently, the generalizability of these findings to frailer populations or those with significant comorbidities is limited. Future longitudinal studies incorporating more diverse samples are warranted to further validate and extend these findings.

## Data Availability

The original contributions presented in the study are included in the article/supplementary material, further inquiries can be directed to the corresponding author.
